# Development of a Smartphone-Integrated Reflective Scatterometer for Bacterial Identification

**DOI:** 10.3390/s22072646

**Published:** 2022-03-30

**Authors:** Iyll-Joon Doh, Brianna Dowden, Valery Patsekin, Bartek Rajwa, J. Paul Robinson, Euiwon Bae

**Affiliations:** 1 Applied Optics Laboratory, School of Mechanical Engineering, Purdue University, West Lafayette, IN 47907, USA; idoh@purdue.edu; 2 Basic Medical Science, College of Veterinary Medicine, Purdue University, West Lafayette, IN 47907, USA; dowdenb@purdue.edu (B.D.); valerythe1@gmail.com (V.P.); jpr@cyto.purdue.edu (J.P.R.); 3 Bindley Bioscience Center, Purdue University, West Lafayette, IN 47907, USA; brajwa@purdue.edu; 4 Weldon School of Biomedical Engineering, Purdue University, West Lafayette, IN 47907, USA

**Keywords:** bacterial identification, biosensor, microbial analysis, portable analytic system, label-free detection, diffraction, light scattering

## Abstract

We present a smartphone-based bacterial colony phenotyping instrument using a reflective elastic light scattering (ELS) pattern and the resolving power of the new instrument. The reflectance-type device can acquire ELS patterns of colonies on highly opaque media as well as optically dense colonies. The novel instrument was built using a smartphone interface and a 532 nm diode laser, and these essential optical components made it a cost-effective and portable device. When a coherent and collimated light source illuminated a bacterial colony, a reflective ELS pattern was created on the screen and captured by the smartphone camera. The collected patterns whose shapes were determined by the colony morphology were then processed and analyzed to extract distinctive features for bacterial identification. For validation purposes, the reflective ELS patterns of five bacteria grown on opaque growth media were measured with the proposed instrument and utilized for the classification. Cross-validation was performed to evaluate the classification, and the result showed an accuracy above 94% for differentiating colonies of *E. coli*, *K*. *pneumoniae*, L. *innocua*, *S. enteritidis*, and *S. aureus.*

## 1. Introduction

The conventional approach to foodborne pathogen detection relies on bacterial cultures. The procedure requires the culturing of the microbial organisms on agar plates with selective media, and it is often followed by secondary biochemical identification [1]. The plate culture method is still regarded as the gold standard, although it is time-consuming, laborious, requires expensive consumables and trained personnel [2]. Therefore, extensive research has been conducted to develop a rapid, reliable, reagent-free, and user-friendly system. We and others have demonstrated optical techniques that utilize the light scattering or diffraction pattern of a bacterial colony grown on agar plates for bacterial identification [3,4,5,6,7,8,9]. The comparison of these technologies are summarized in the Supplementary Materials (Table S1). The advantage of these techniques is that they are label-free and non-destructive, so the data collection can be executed along with the conventional plate culturing. This complements the existing plate culture method by shortening the process of selective or differential plating, saving cost and time during identification. The elastic light scattering (ELS) technique which creates the light scattering pattern by illuminating the colony with a coherent and collimated light source, has shown a robust performance with a variety of foodborne pathogens. However, the ELS technique employing a forward light scattering pathway cannot be used when the growth media is opaque (such as blood or chocolate agar) or the colony is optically dense. Therefore, a reflective (backscattering) ELS technique was introduced to differentiate the colonies grown in such conditions. The feasibility was tested on four different bacteria, resulting in over 95% of classification accuracy [9]. Because of the wide reflection angle, instead of a lensless image sensor, a high-resolution DSLR (digital single lens reflex) camera and a screen were utilized to capture patterns. This design choice affected the size of the instrument. Despite the simple setup, the instrument required 279.4 mm (L) × 355.6 mm (W) × 490 mm (H) of space. 

Recent advancements in smartphone design have changed the landscape of analytic platforms for food safety, opening up the opportunity to develop portable, field-deployable, and inexpensive analytic devices. The traditional benchtop analytic instruments are bulky, heavy, expensive, and incompatible with on-site detection. Smartphone-based devices have emerged as alternatives to conventional analysis tools [10]. For example, Muller et al. developed a smartphone-based fluorescence microscope to detect pathogenic bacteria in complex samples and showed a detection limit comparable to a conventional microscope [11]. Min et al. demonstrated a field-deployable smartphone-based lateral flow assay (LFA) analyzer that provided quantitative analysis and enhanced the sensitivity of commercial LFA rapid kits [12]. Furthermore, smartphones are also widely applied in other types of optical sensing methods such as bioluminescence [13,14], colorimetric [15,16], and spectroscopic detections [17,18]. They are also integrated into the electrochemical sensing platforms as amperometric, potentiometric, and impedimetric biosensors [19,20]. The cited works demonstrated that the use of smartphones as control and storage devices for various biosensors enables the development of portable and user-friendly analytical systems. Although the performance of the smartphone-based instruments does not equal the one offered by the benchtop systems yet, owing to operability, connectivity, portability, and the presence of built-in sensors, smartphones have become ideal control, interaction, and analysis tools in on-site sensing systems [10]. 

This paper presents a newly developed bacterial identification device using the reflective ELS technique. The instrument utilizes a screen and a camera like the previous design, but instead of the DSLR camera, a smartphone was used to reduce the overall size of the instrument. The device’s chassis was built with a 3D printer, and a green diode laser was utilized to generate the backscatter pattern. Owing to the incorporation of a smartphone, the proposed device was not only reduced in size but also became much easier to operate. For the validation of the method, five major bacteria genera, *Escherichia coli*, *Klebsiella pneumoniae*, *Listeria innocua*, *Salmonella enteritidis*, and *Staphylococcus aureus*, were tested; the reflective ELS patterns of their colonies were acquired and classified to evaluate the performance of the device.

## 2. Materials and Methods

### 2.1. Instrument Design

**Figure 1:**
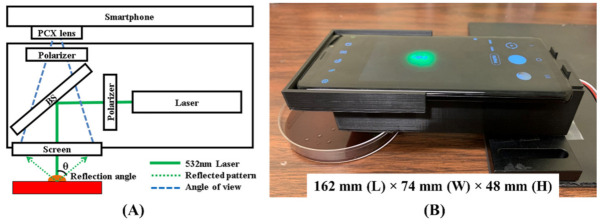
Smartphone-based reflective bacterial colony phenotyping device. (**A**) A schematic illustration of reflective ELS pattern generation with the proposed instrument. (**B**) A picture of the instrument measuring reflective ELS pattern of a colony on BHI with 5% horse blood agar plate.

Figure 1 demonstrates the schematic diagram and a picture of the smartphone-based bacterial colony phenotyping instrument. The chassis of the device was built with a black PLA material using a 3D printer (Ultimaker, Framingham, MA, USA). The frame’s inner surface was covered with a non-reflective material to avoid any stray light interfering with the reflection patterns of the bacterial colony. A 5 mW, 532 nm diode-pumped solid-state (DPSS) point laser (#37-027, Edmund Optics, Barrington, NJ, USA) was utilized as the light source. A 50:50 beam splitter (BS) (#31-436, Edmund Optics, Barrington, NJ, USA) was placed to refract the incoming beam towards the bacterial colony. A rear projector film was placed below the BS, a screen material to project the pattern. A 2 mm diameter hole was drilled in the screen and aligned with the laser beam to let the beam travel through the screen, as illustrated in Figure 1A. On the other side of the BS, the smartphone with 12 megapixel imaging sensor was placed to capture the pattern formed on the screen. The smartphone, the Samsung Galaxy S9 model (SM-G960U), provided a manual mode that enabled the manual control of the camera setting, such as exposure, aperture, focus, etc. Therefore, the reflected patterns were collected in the same measurement conditions throughout the experiment. The exposure time, aperture, and ISO were fixed to 1/10 s, f/1.5, and ISO-320. An *f* = 50 mm plano-convex lens (LA1213-A, Thorlabs Inc., Newton, NJ, USA) was mounted in front of the smartphone camera to adjust the focus. A pair of the polarizers (#54-926, Edmund Optics, Barrington, NJ, USA) was used to minimize the intensity of the specular reflection directly coming from the colony. Because of the specular reflection, the direct reflection of the laser beam caused noise and saturation to the pattern image, a clear pattern was difficult to measure when the smartphone camera, the hole in the screen, and the colony were in perfect alignment. Thus, they were slightly off-axis to prevent the specular reflection from directly illuminating the image sensor. The overall dimension of the device was 162 mm (L) × 74 mm (W) × 48 mm (H), including the smartphone. Additional pictures documenting the components of the proposed instrument are attached in the Supplementary Materials (Figure S1). In Figure 1A, the optical path is illustrated. The incident laser beam is represented with the green solid line, labeled as “532 nm laser” whereas the reflected light from the colony is illustrated by the dotted green line, labeled as “Reflected pattern”. The dashed blue line represents the view angle from the camera. The diagram shows that the smartphone camera views through the beamsplitter and captures the pattern formed on the rear projection screen.

### 2.2. Sample Preparation

The proof-of-concept experiments, phenotyping five bacterial species, were conducted, and *Escherichia coli*, *Klebsiella pneumoniae* ATCC 13887, *Listeria innocua*, *Salmonella enteritidis* ATCC 13076, and *Staphylococcus aureus* were prepared on opaque agar media. *E. coli*, *L. innocua*, and *S. aureus* were confirmed with PCR test using the primers for ATCC strain bacteria such as *E. coli* ATCC 25922, L. *innocua* ATCC 33090, and *S. aureus* ATCC 23235 for the reference. These bacteria genera are some of the most popular foodborne pathogens, which outbreak as a contaminant to vegetables, mushrooms, and meats. All cultures were subcultured from −80 °C freezer stored stock cultures and streaked on trypticase soy agar (TSA) (Bacto, BD Diagnostics, Franklin Lakes, NJ, USA). The streak plates were incubated at 37 °C until the colonies were visible. One colony from each organism culture was picked and serially diluted in 4 mL buffer solution (PBS) three times by a factor of 1:40. A 50 μL aliquot of the last dilution tube was spread on the opaque media plate using an L-shaped sterile spreader (2910, Globe Scientific, Mahwah, NJ, USA) and the dish was incubated at 37 °C until the diameter of the colony reached 50–1000 µm. The diameter of the colony was controlled to have a comparable size of the reflection pattern across the species. Opaque media, brain heart infusion (BHI) agar with 5% horse blood, buffered charcoal yeast extract (BCYE) agar (BBL, BD Diagnostics, Franklin Lakes, NJ, USA), chocolate agar (3P-125, Edge Biologicals Inc., Memphis, TN, USA), and charcoal blood agar with cephalexin (R01298, Thermo Fisher Scientific, Waltham, MA, USA) were prepared to investigate the reflective ELS patterns of the sample bacteria and explore the change of the patterns with respect to the nutrition media. The culture media were selected based on their application and opacity. The blood agar and chocolate agar media are two of the most popular non-selective media that are opaque whereas charcoal and BCYE agar are selective (differential) media that are opaque and widely used in the clinical area for Gram-negative species.

### 2.3. Measurement and Classification

**Figure 2:**
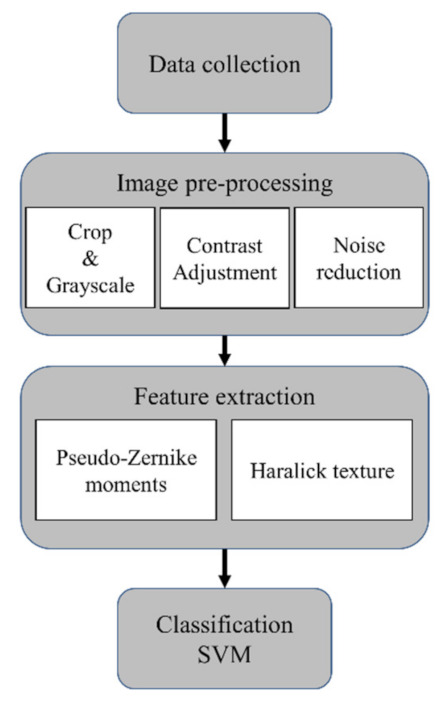
The reflective ELS pattern measurement procedure’s flow chart includes data collection, image pre-processing, feature extraction, and classification with a machine-learning algorithm.

Figure 2 shows the flow chart of the bacterial identification using the new reflective ELS-based setup. A Petri dish with bacterial colonies was placed underneath the device aligning the colony to the laser beam to collect the pattern images. The reflected pattern was then formed on the screen and captured using the camera app on the smartphone. The collected pattern images were saved in the smartphone’s internal storage and then transferred to the PC for image processing and classification. Owing to the wireless capability of the smartphone, the collected data can be uploaded to online storage for easy access to the data files from the PC. The scatter patterns represented as bitmap images were pre-processed with Matlab to enhance the contrast. The patterns were first saved as grayscale, cropped to a square image with 1024 × 1024 pixels, adjusted for contrast. Next, the noise reduction process followed to remove speckles. Figure S2 displays the output image of each step to show the change of the reflective ELS pattern along with the image pre-processing. For the classification, the processed images were loaded to Baclan Software, a stand-alone quantitative image processing software incorporating feature extraction and classification [9,21]. Following the procedures described in our previous studies, two groups of features were extracted: pseudo-Zernike moments and Haralick texture features [9,22]. Pseudo-Zernike moments are invariant orthogonal moments computed using pseudo-Zernike polynomials. They capture the characteristics of circular and uniform patterns. Haralick texture features were included to increase the feature space, contributing to a more accurate recognition of disordered patterns. A classification model was constructed using a support vector machine (SVM) algorithm. A 10-fold cross-validation (CV) with a 20-times repetition was performed to calculate the accuracy, sensitivity, specificity, positive predictive value (PPV), and negative predictive value (NPV).

## 3. Results

### 3.1. Reflection Pattern Images

**Figure 3:**
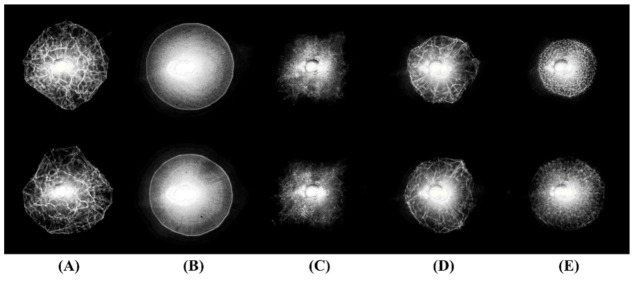
Representative reflective ELS patterns of the five bacteria cultured on BHI with 5% horse blood agar: (**A**) *E. coli*, (**B**) *K. pneumoniae*, (**C***) L. innocua*, (**D**) *S. enteritidis*, and (**E**) *S. aureus*. The two rows show multiple colonies analyzed for each analysis.

The reflective ELS patterns of the five bacteria grown on BHI with 5% horse blood agar were collected using the proposed device and processed to enhance the contrast of the pattern. After the image processing, patterns were sharper, and their unique characteristics became more apparent. Without the reflective ELS patterns, colonies were not easily differentiable with the naked eye because their morphological characteristics, such as form, margin, and pigmentation were not apparent at the particular size. In Figure 3, the representative pattern images of the five bacteria are presented for qualitative comparison, given in two rows to demonstrate the repeatability. Based on the visual inspection, each species showed unique and distinguishable characteristics in their reflective pattern images. A bright spot at the center is the laser beam spot shown through the hole. Because the hole in the screen was slightly larger than the beam spot and closer to the camera, dark rings were observed around the beam spot. The colonies of *E. coli* (Figure 3A) and *S. enteritidis* (Figure 3D) produced patterns with similar-looking shapes with non-uniform patterns at the inner area and noncircular outer edge. However, patterns from *S. enteritidis* (Figure 3D) demonstrated clear radial spokes, whereas *E. coli* (Figure 3A) did not show this feature. The *L. innocua* patterns (Figure 3C) also had irregular shapes, but the peripheral area of its patterns differed from the patterns from *E. coli* and *S. enteritidis* (Figure 3A,D); the patterns were not enclosed by borderlines, and the boundary was unclear. The patterns from *K. pneumoniae* (Figure 3B) were formed with densely packed concentric rings, and had the highest overall intensity, as their colonies were more reflective, resulting in a greater saturated area at the center of the pattern. On the other hand, *S. aureus* (Figure 3E) also had circular shape patterns, but distinctive patterns were observed in the inner area of the pattern.

### 3.2. Genera Level Classification

For each class, 50 reflective ELS pattern images were prepared for feature extraction, and the combination of pseudo-Zernike moments and Haralick texture features were employed to describe the patterns. The order of the pseudo-Zernike moments was set to 20, while the distance and the number of levels were set to 1 and 64, respectively, for Haralick texture. The total number of features extracted from each pattern image was 243, where 13 of them were from Haralick texture, and the others were from pseudo-Zernike moments. As a result of the classification, the CV matrix was obtained from an SVM-based classifier. The performance was evaluated by five criteria: accuracy, sensitivity, specificity, PPV, and NPV. These statistical parameters were calculated based on the CV matrix and provided in Table 1 to show the differentiability of the five bacteria using the reflective ELS patterns captured by the proposed instrument. The CV result was promising as every class had all of the accuracy measures greater than 96%. The CV matrix had all the diagonal elements that resulted in values greater than 95% (Table S2). The visual inspection of the patterns demonstrated that each organism produced a unique and distinct pattern shape; the classification result also showed excellent separations between the classes.

### 3.3. Colonies on Various Nutrition Media

**Figure 4:**
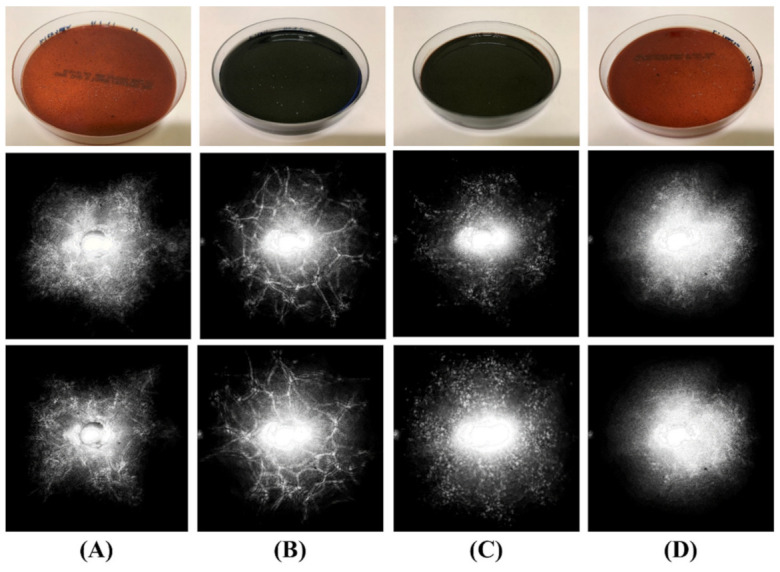
Reflected ELS pattern images of *L. innocua* colonies cultured on (**A**) BHI with 5% horse blood, (**B**) BCYE, (**C**) charcoal, and (**D**) chocolate agar with the pictures of their petri dish.

The five bacteria were also plated on BCYE, charcoal, and chocolate agar. Their colonies were interrogated using the proposed instrument to explore the change of reflective ELS patterns with respect to the type of nutrition media. Again, the shape of each reflected pattern was qualitatively compared to observe any differences induced from individual media. The reflective ELS patterns of *L. innocua* colonies plated on BHI blood agar, BCYE agar, charcoal agar, and chocolate agar are presented in Figure 4 for comparison. The effect of the nutrition media on the pattern’s shape was apparent because the patterns formed differently although they were the same species. On BHI agar with 5% horse blood (Figure 4A), the *L. innocua* colonies were not uniform or ring type patterns, and diffractions were randomly organized. However, the colonies on BCYE agar (Figure 4B) produced patterns with more definite diffraction lines, while the others were more likely to have randomly scattered light patterns. The patterns from the chocolate agar (Figure 4D) had higher intensity than the others, as most areas of the pattern were saturated. The reflective ELS patterns of the other bacteria grown on BCYE, charcoal, and chocolate agar are also provided in the Supplementary Materials (Figure S3). 

For each nutrition media, the classification was performed to differentiate the five bacteria using their reflective ELS patterns. The CV matrices are provided in the Supplementary Materials (Table S2), and the classification performance based on the five statistical criteria is presented in Table 2. The same type of features and classifiers were employed for the classification. Because some species did not grow on the charcoal agar, the classification result for charcoal agar was excluded to maintain an equal number of classes for comparison since a different number of classes could cause bias the outcomes. The classification results of the particular case are separately presented in the Supplementary Materials (Table S3), where the five bacteria were again nicely classified regardless of the type of nutrition media, showing that the diagonal elements of the CV matrix for the BCYE agar case were all greater than 83%. At the same time, they were all over 85% for chocolate agar. In Figure S3, although some patterns showed similarity in their texture or shape and were difficult to differentiate with the naked eye, they were classified as different patterns when proper descriptive features were incorporated for the classification.

## 4. Discussion

### 4.1. Hardware Design

**Figure 5:**
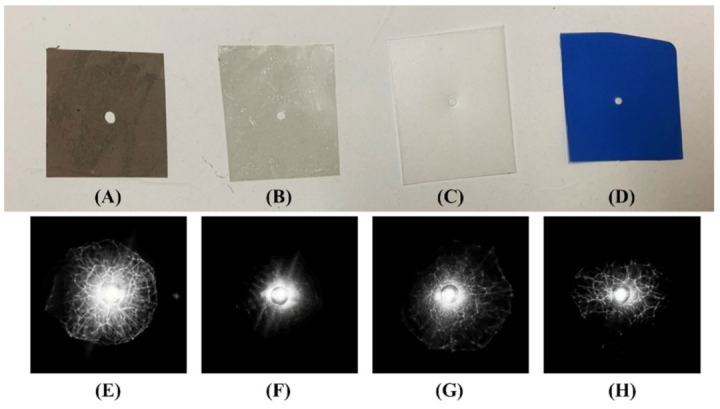
The tested screen materials and the reflected ELS patterns created on the corresponding screen. The picture of the screen materials shows (**A**) the rear projection screen material (dark), (**B**) the rear projection screen material (transparent), (**C**) diffusive material (white), (**D**) diffusive material (blue). The reflective ELS patterns of *E. coli* on (**E**) rear projection screen material (dark), (**F**) rear projection screen material (transparent), (**G**) diffusive material (white), and (**H**) diffusive material (blue).

The major innovation from the previous design was that the device’s size was dramatically reduced and it became a handheld detection device. The height of the new device was about 10% of the previous size, which employed a high-resolution DSLR camera, a personal computer, and a large screen to capture the reflective ELS pattern [9]. In the earlier setup, the screen was located above the beam splitter, which led to a longer distance (140 mm) between the colony and the screen. Thus, the reflective ELS pattern on the screen was too large because the DSLR camera had to be 350 mm above the screen. For some colonies, the half cone reflection angle was greater than 45°, which caused the shadow of the beam splitter to interfere with the reflected pattern, and this was unavoidable when the BS was located in between the colony and screen. In the new design, the screen was placed below the beam splitter, close to the colony. The distance between the screen and the camera was also shortened, and the DSLR camera was replaced with a smartphone. The overall height was reduced from 490 mm to 48 mm. Because the screen was in the middle of the laser beam path, a hole was punched in the screen to allow laser beam passage. The hole in the screen did not affect the quality of the delete let patterns because the center of the reflective ELS pattern was a specularly reflected incident beam that saturated the sensor, and the majority of the information lay in the peripheral area. One advantage of integrating the screen and camera was that this was more cost-effective than utilizing an image sensor since the reflective system required a large active area. To select the best screen material, an experiment was conducted with four types of screen material. Two rear projection films with different transparencies and thin diffusive materials with two different colors, gray and blue, were examined. The clearest pattern was formed on the dark rear projection film (Figure 5A), as shown in Figure 5E, while the transparent rear projection film (Figure 5B) could not pick up the pattern in Figure 5F. The patterns formed on the other screen materials were visible but not as differentiated as those on the dark rear projection film. Moreover, they were disturbed by the screen’s texture because the diffusive materials did not have a perfectly smooth surface. Thus, a dark rear projection film was selected for the screen.

One of the most significant advantages of the smartphone-integrated analytic device is that the smartphone already has a variety of built-in sensors. Among the sensors, the imaging sensor is by far the most commonly incorporated in smartphone-based devices as an optical transducer to capture the optical signals from biological or chemical reactions. Accordingly, a calibration process was necessary due to significant variation of the color profiles between different smartphones because spectroscopic or colorimetric measurements are sensitive to the spectral responsiveness of the image sensor [23]. Nevertheless, for the proposed device, variation of the smartphone is unlikely to affect the quality of the pattern image unless the camera resolution is significantly lower. This is because the classifying features are mainly extracted from the shape or intensity profile of the pattern rather than the image’s color profile. Therefore, our device is not restricted to a specific smartphone model. Another advantage is that the smartphone-based system has its own operating system and computing power, where the data processing or analysis can be done with a smartphone app. Given that wireless network supply is limited in most rural or developing areas, a robust and reliable smartphone app is required for on-site and rapid target quantification and analysis [24]. Thus, many smartphone-based systems have been proposed with their custom-built apps to process the image and analyze the data without assistance from a computer [25,26]. Although current analysis with a smartphone app is not as powerful as traditional computer processing at this time, continuing improvement will result as smartphone computing power improves. Further investigation is necessary to develop a well-established smartphone app for reflective ELS pattern analysis, to establish a completely stand-alone unit.

### 4.2. Colony Morphology and Reflective Scattering Pattern

One of the critical factors of the bacterial colony was the aspect ratio—a ratio of colony elevation to the diameter. According to our previous study, the aspect ratio and the reflection angle were closely related as they were positively correlated [9]. It was essential to keep the colony size with optimal aspect ratio, and therefore, the diameter of the colony was not fixed across the species but controlled to have similar size patterns on the screen. *K. pneumoniae* and *S. aureus* colonies on BHI with 5% horse blood agar had enormously large reflection angles whose patterns exceeded the screen area when their colony diameters were around 800–1000 μm. Generally, the aspect ratio of the colony gradually increased along with the growth and plateaued once it reached the maximum [27]. Therefore, to keep the patterns smaller than the screen area, the bacteria were measured while their colonies were actively growing. The classification result proved that the reflective ELS patterns of small colonies whose diameters were around 500–800 μm were precisely classified. There were also cases that the reflection patterns were already too large even though the colonies were still young and small. *K. pneumoniae* already had their colonies with extremely high aspect ratios when they were grown on BCYE and charcoal agar plates, resulting in very large patterns on the screen. Moreover, the reflected pattern intensity was not adequate, and consequently, full patterns were not measured for these cases, but only the center portions of the patterns were captured. The reflectivity of the colony and the nutrition media was another factor that affected the quality of the pattern. Highly reflective colonies or transparent colonies that affect more reflectance at the surface of agar generate the reflective ELS patterns with higher intensities as shown in Figure 3B and Figure 4D. *K. pneumoniae* colonies on BHI 5% horse blood agar were mucoid colonies whose surface was highly reflective whereas *L. innocua* colonies on chocolate agar were translucent.

The different types of growth media affected the shape of the reflective ELS pattern. Similar studies were conducted by Bae et al. to examine the correlation between forward ELS patterns and nutrition concentration. They showed that the colony morphology and the forward ELS patterns were highly related to the nutritional concentration of the media [28]. As reflected patterns are also closely related to the colony morphology, the shapes of the reflected patterns were expected to change with respect to the type of media. The comparison of *L. innocua* patterns from various nutrition media, presented in Figure 4, showed that they were clearly different in terms of shape. This was confirmed by running the pattern images on the machine learning classifier with their pseudo-Zernike moments and Haralick texture features. Based on the *L. innocua* patterns, the classification of the four nutrition media output all the diagonal elements of CV matrix resulting in 100%, implying that the media type induced the difference in the colony morphology, and their differences were clearly and precisely measured through the reflective ELS patterns using the proposed instrument.

Despite being the same species, a mutation can create a difference in the colony morphology that results in different shapes of the reflective ELS patterns. As shown in Figure S4, a completely different shape of the pattern was observed when the colonies were mucoid or non-mucoid. The distinctive morphological characteristics were directly portrayed in the pattern. Compared to non-mucoid colonies, the mucoid colony had a smoother and more reflective colony surface. Therefore, the shape of the reflective ELS patterns in Figure S4A was completely different from the patterns in Figure S4B. Figure S4A had the patterns with uniform and concentric rings whereas the patterns in Figure S4B were divided into two sections. Moreover, owing to the highly reflective surface, Figure S4A had a greater overall intensity than Figure S4B. This demonstrates that the reflective ELS pattern-based method can also distinguish the mutant strains. The ELS pattern-based technique does not require any labels but requires a pattern image library to identify an unknown organism. Therefore, as long as the ELS pattern information of the mutant strain is stored in the database, this technique can distinguish and identify the mutant colonies. 

### 4.3. Classification

**Figure 6:**
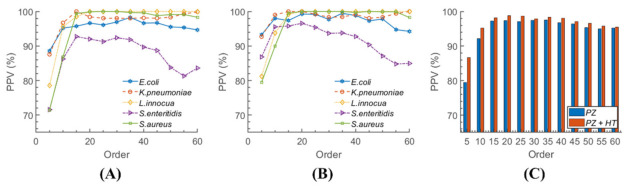
The PPVs of the five bacteria with respect to the pseudo-Zernike polynomial order. The feature set comprises (**A**) only pseudo-Zernike moments and (**B**) pseudo-Zernike moments with Haralick textures. (**C**) Compares average PPVs of each feature set.

The feature set was composed of Haralick texture descriptors and pseudo-Zernike moments. Haralick features were selected based on their successful use in our previous reports, where they provided an increase in classification efficiency when applied to the reflective ELS patterns [9]. We opted to use pseudo-Zernike moments instead of Zernike moments as they are more robust and less sensitive to image noise [22]. The preferred order of pseudo-Zernike polynomials was selected through iterative testing from 5 to 60 with an increment of 5. The result suggested that the feature set with a pseudo-Zernike polynomial order of 20 delivered the best overall accuracy, sensitivity, specificity, PPV, and NPV. Although the dimensions of the feature set increased exponentially as the order increased, the higher number of features did not ensure a better classification rate. This is shown in Figure 6, which demonstrates the PPVs of each organism with respect to the pseudo-Zernike polynomial order. The PPVs started to increase as the order increased, but they decreased along with the polynomial order after reaching the maximum. This implies that a naïve increase in the feature dimensionality could result in overfitting, and consequently, a simple lower practical classification performance. Therefore, a process of finding the optimal polynomial order is necessary for acceptable classification results. Figure 6 also shows that the Haralick texture features generally improved the classification performance since the PPVs in Figure 6B were mostly greater than the corresponding PPVs in Figure 6A. A direct comparison is given in Figure 6C, where each bar represents the average PPV of the five bacteria. It is more apparent in Figure 6C that the combined feature set resulted in higher PPVs than when using pseudo-Zernike alone. This again illustrates that Haralick texture features had an important role in differentiating reflective ELS patterns. They are particularly important for classifying disordered and web-like shapes, unlike the circular and uniform patterns formed by forward ELS patterns. 

## 5. Conclusions

A smartphone-based bacterial colony phenotyping instrument using a reflective ELS pattern was developed. Owing to the new design incorporating the smartphone as a control and data storage unit, the overall size of the instrument was significantly decreased, which enhanced portability and lowered the cost of foodborne pathogen detection. For the validation, five bacterial genera were interrogated. The classification was performed using the combination of descriptors, including pseudo-Zernike moments and Haralick texture features. The genera level classification resulted in excellent differentiability between the bacteria: accuracies exceeding 98%, 97%, and 94% for the colonies on BHI with 5% horse blood, BCYE, and chocolate agar, respectively. The classification performance, robustness, and repeatability of the measurements encourage further investigation of the smartphone-based ELS system and testing with other species of bacteria. An obvious next step is the development of a smartphone classification app that would perform all the functions delivered currently by the offline external Baclan software package. This arrangement would allow for a fully independent smartphone-based reflective ELS system.

## Figures and Tables

**Table 1:** Classification of the five bacteria grown on BHI with 5% horse blood agar using the reflective ELS patterns of their colonies. The results are presented in accuracy, sensitivity, specificity, PPV, and NPV.

	*E. coli*	*K. pneumoniae*	*L. innocua*	*S. enteritidis*	*S. aureus*
Accuracy	98.8	100	100	98.8	100
Sensitivity	97.1	100	100	96.7	100
Specificity	99.2	100	100	99.3	100
PPV	96.7	100	100	97.1	100
NPV	99.3	100	100	99.2	100

**Table 2:** Classification results of the five bacteria grown on BCYE (top) and chocolate agar (bottom) using the reflective ELS patterns of their colonies. The results are presented in accuracy, sensitivity, specificity, PPV, and NPV.

	*E. coli*	*K. pneumoniae*	*L. innocua*	*S. enteritidis*	*S. aureus*
BCYE Agar					
Accuracy	94.8	98.7	95.4	99.6	97.7
Sensitivity	83.9	93.7	90.0	97.9	100
Specificity	97.6	100	96.7	100	97.1
PPV	89.5	100	87.3	100	89.6
NPV	96.0	98.5	97.5	99.5	100
Chocolate Agar					
Accuracy	94.6	96.8	99.8	96.0	97.8
Sensitivity	90.5	90.4	98.9	85.5	97.2
Specificity	95.6	98.5	100	98.6	97.6
PPV	83.8	93.6	100	94.0	92.1
NPV	97.6	97.6	99.7	96.5	99.4

## Data Availability

Not applicable.

## Supplementary Materials

The following supporting information can be downloaded at: https://www.mdpi.com/article/10.3390/s22072646/s1. Figure S1. More pictures of the proposed instrument. (A) Pictures showing each component of the proposed instrument, (B) A picture of the instrument without the smartphone and top cover, measuring the reflective ELS pattern of E. coli colony on BHI with 5% horse blood agar. Figure S2. Image pre-processing procedure and corresponding setting or MATLAB functions utilized. Example pattern images showing the changes made from each step are attached. Figure S3. Representative reflective ELS pattern images of the five bacteria grown on various nutrition media. The rows represent the media type, and the columns represent the bacterial species. Figure S4. The comparison between mucoid and non-mucoid bacterial colonies with their reflective ELS patterns. The reflective ELS patterns of K. pneumoniae that formed (A) mucoid and (B) non-mucoid colonies. Table S1. List of the optical scattering/diffraction technologies for bacterial identification and their specifications. Table S2. The cross-validation matrices show the classification result of the five bacteria grown on BHI with 5% horse blood, BCYE, and chocolate agar plates. Table S3. The cross-validation matrix and the five statistical parameters for the four bacteria grown on charcoal agar plates. Since S. aureus did not grow on this media, only four bacteria were classified. References [3,4,5,6,8,9] are cited in the Supplementary Materials.
