# Supplementary material for: Development of a Smartphone-Integrated Reflective Scatterometer for Bacterial Identification
Source: Sensors (Basel). 2022 Mar 30;22(7):2646. doi: 10.3390/s22072646 (PMC9003293; doi:10.3390/s22072646)
Supplement: Supplementary file 1 [file sensors-22-02646-s001.zip › sensors-1612204-supplementary.pdf]

Article

Development of a Smartphone-Integrated Reflective Scatterometer for Bacterial Identification

Iyll-Joon Doh [1], Brianna Dowden [2], Valery Patsekin [2], Bartek Rajwa [3], J. Paul Robinson [2,4] and Euiwon Bae [1,*]

[1] Applied Optics Laboratory, School of Mechanical Engineering, Purdue University, West Lafayette, IN 47907, USA; idoh@purdue.edu
[2] Basic Medical Science, College of Veterinary Medicine, Purdue University, West Lafayette, IN 47907, USA; dowdenb@purdue.edu (B.D.); valerythe1@gmail.com (V.P.); jpr@cyto.purdue.edu (J.P.R.)
[3] Bindley Bioscience Center, Purdue University, West Lafayette, IN 47907, USA; brajwa@purdue.edu
[4] Weldon School of Biomedical Engineering, Purdue University, West Lafayette, IN 47907, USA
* Correspondence: ebae@purdue.edu

Supplementary Materials:

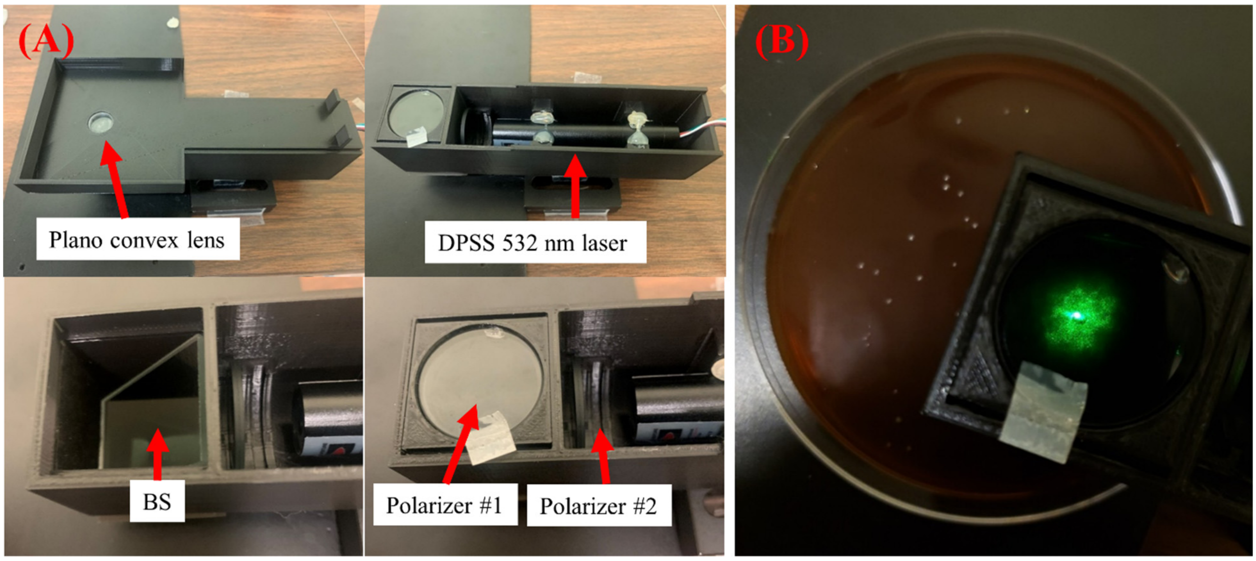

Figure S1. More pictures of the proposed instrument. (**A**) Pictures showing each component of the proposed instrument, (**B**) A picture of the instrument without the smartphone and top cover, measuring the reflective ELS pattern of E. coli colony on BHI with 5% horse blood agar.

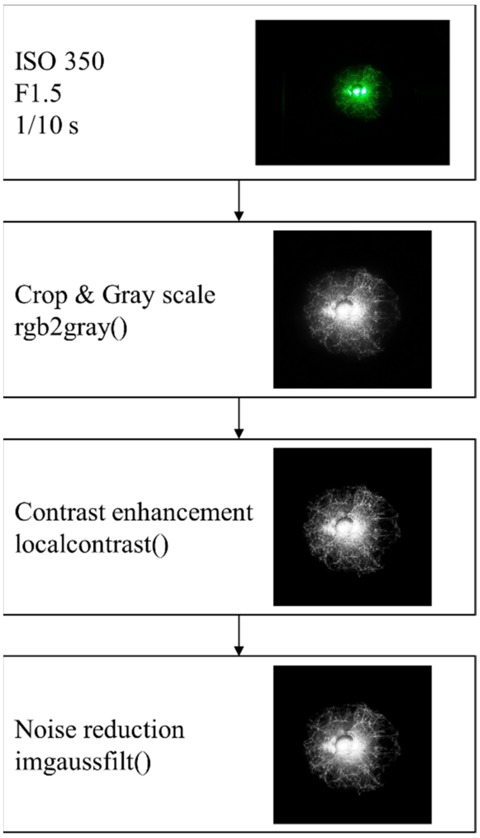

Figure S2. Image pre-processing procedure and corresponding setting or MATLAB functions utilized. Example pattern images showing the changes made from each step are attached.

Matlab function, "rgb2gray(RGB)" converts the RGB image to grayscale image by eliminating the hue and saturation information while retaining the luminance. "localcontrast()", is an edge-aware local contrast manipulation of images, which enhances or flattens the local contrast of an image by increasing or smoothing details while leaving strong edges unchanged. "imgausfilt()" is a 2-D Gaussian filtering function that filters an image with a 2-D Gaussian smoothing kernel with specified standard deviation. Details of the Matlab functions can be found on Matlab website.

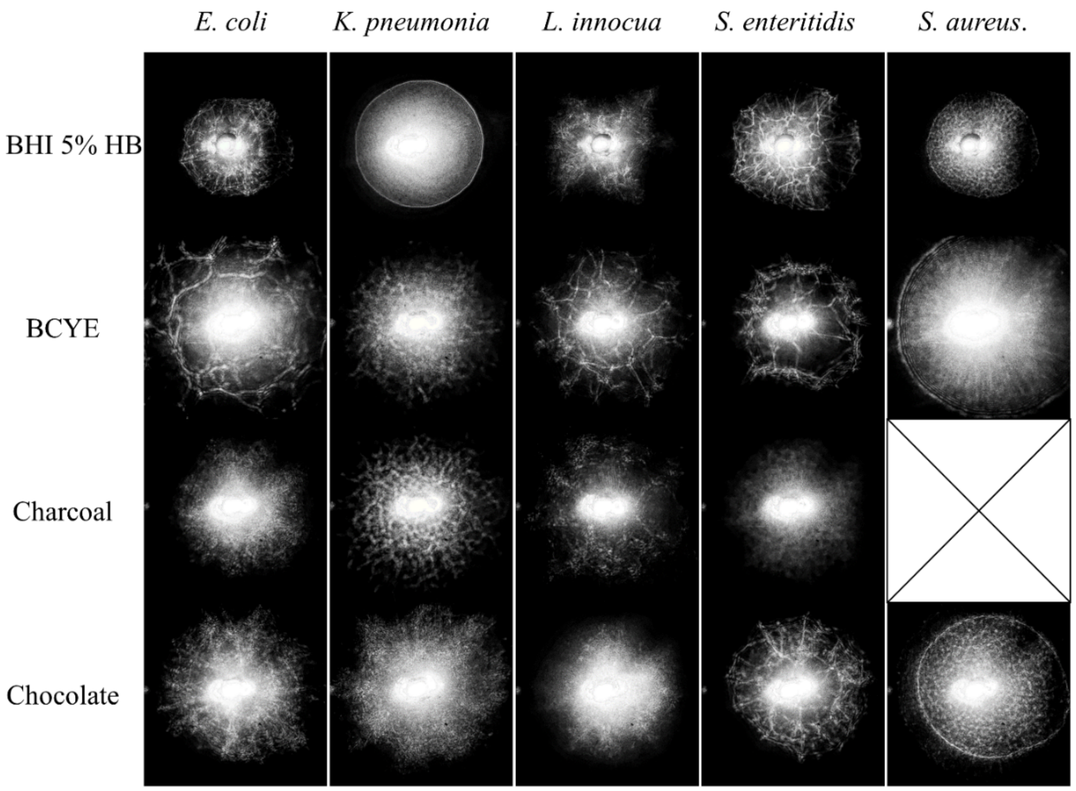

Figure S3. Representative reflective ELS pattern images of the five bacteria grown on various nutrition media. The rows represent the media type, and the columns represent the bacterial species.

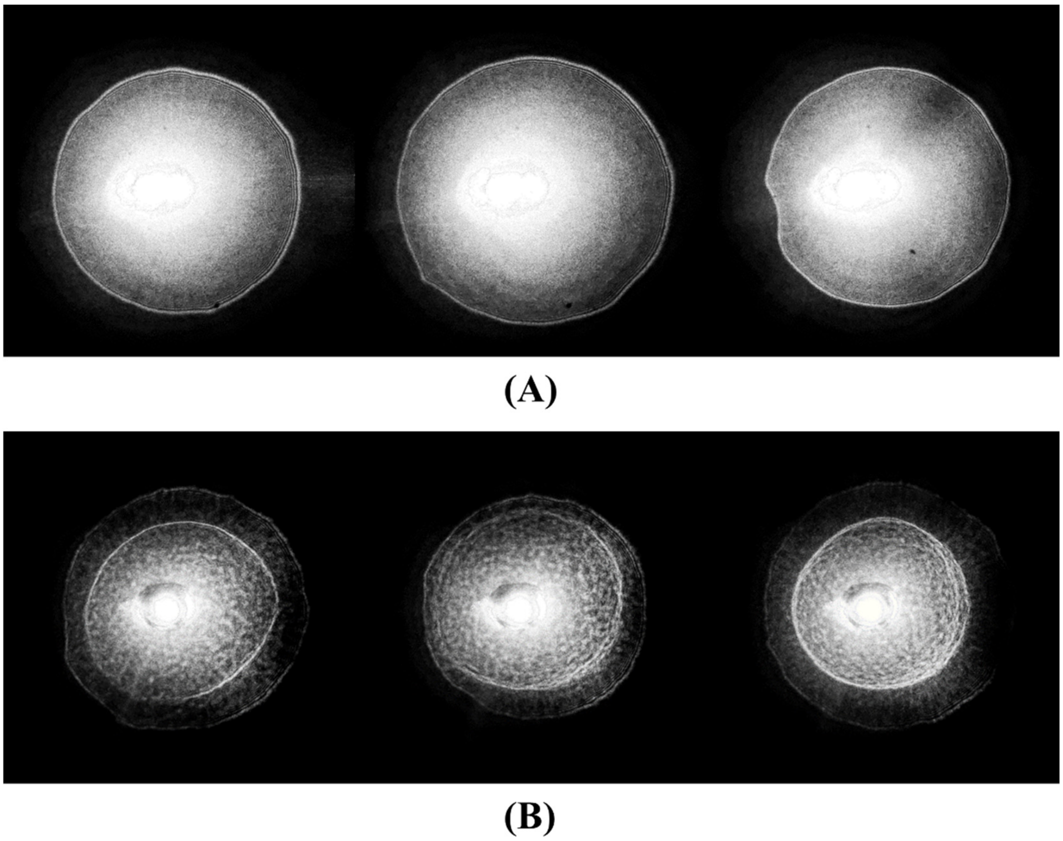

Figure S4. The comparison between mucoid and non-mucoid bacterial colonies with their reflective ELS patterns. The reflective ELS patterns of *K. pneumoniae* that formed (**A**) mucoid and (**B**) non-mucoid colonies.

Table S1. List of the optical scattering/diffraction technologies for bacterial identification and their specifications.

	Laser wavelength	Pattern type	Target sample	Detector	Features	Classifier	Ref.
Bae et al	635 nm	Transmitted	Foodborne pathogen	CMOS	Haralick texture<br>Zernike [illegible]	SVM	[3]
Marcoux et al	543.5 nm	Transmitted	Clinical	CCD	Zernike invariant	Bayes Network<br>Naïve Bayes<br>SMO	[4]
Minoni et al	Not specified	Transmitted	Urinary tract pathogen	Not specified	NA	NA	[5]
Buzalewicz et al	635 nm	Transmitted	Clinical	CCD	Mean<br>Entropy<br>Radius<br>Standard deviation<br>Flatness<br>Skewness	LDA<br>QDA<br>SVM	[6]
Pan et al	980 nm	Transmitted	Foodborne pathogen	Screen<br>NIR camera	Zernike invariant	LDA<br>KNN<br>PLSDA<br>BPANN<br>[illegible]	[8]
Kim et al	532 nm	Reflected	Foodborne pathogen	Screen<br>DSLR camera	Haralick texture<br>Zernike invariant	SVM	[9]

Table S2. The cross-validation matrices show the classification result of the five bacteria grown on BHI with 5% horse blood, BCYE, and chocolate agar plates.

BHI 5% HB	*E. coli*	*K.pneumoniae*	*L. innocua*	*S. enteritidis*	*S. aureus*
E. coli	97.1	0	0	2.9	0
K. pneumonia	0	100	0	0	0
L. innocua	0	0	100	0	0
S. enteritidis	3.3	0	0	96.7	0
S. aureus	0	0	0	0	100
BCYE	***E. coli***	***K.pneumoniae***	***L. innocua***	***S. enteritidis***	***S. aureus***
E. coli	83.9	0	9.2	0	6.9
K. pneumonia	0	93.7	1.8	0	4.5
L. innocua	9.8	0	90	0	0.2
S. enteritidis	0	0	2.1	97.9	0
S. aureus	0	0	0	0	100
Chocolate	***E. coli***	***K.pneumoniae***	***L. innocua***	***S. enteritidis***	***S. aureus***
E. coli	90.6	0.8	0	3.8	4.9
K. pneumonia	8.7	90.4	0	0.9	0
L. innocua	0	0.4	98.9	0.1	0.6
S. enteritidis	6.7	5	0	85.5	2.8
S. aureus	2.1	0	0	0.7	97.2

Table S3. The cross-validation matrix and the five statistical parameters for the four bacteria grown on charcoal agar plates. Since *S. aureus* did not grow on this media, only four bacteria were classified.

	E. coli	***K.pneumoniae***	***L. innocua***	***S. enteritidis***
E. coli	90.9	0	9.1	0
K. pneumonia	0	100	0	0
L. innocua	0	0.1	99.9	0
S. enteritidis	0	0	9.6	90.4
	E. coli	***K.pneumoniae***	***L. innocua***	***S. enteritidis***
**Accuracy**	97.7	100	95.3	97.6
**Sensitivity**	90.9	100	99.9	90.4
**Specificity**	100	100	93.8	100
**PPV**	100	99.9	84.2	100
**NPV**	97.1	100	100	96.9
